# Assessing the Content Validity, Acceptability, and Feasibility of the Hypo-METRICS App: Survey and Interview Study

**DOI:** 10.2196/42100

**Published:** 2023-09-29

**Authors:** Uffe Søholm, Natalie Zaremba, Melanie Broadley, Johanne Lundager Axelsen, Patrick Divilly, Gilberte Martine-Edith, Stephanie A Amiel, Julia K Mader, Ulrik Pedersen-Bjergaard, Rory J McCrimmon, Eric Renard, Mark Evans, Bastiaan de Galan, Simon Heller, Christel Hendrieckx, Pratik Choudhary, Jane Speight, Frans Pouwer, Hypo-RESOLVE consortium

**Affiliations:** 1 Medical & Science, Patient Focused Drug Development, Novo Nordisk A/S Søborg Denmark; 2 Department of Psychology, University of Southern Denmark Odense Denmark; 3 Department of Diabetes, School of Cardiovascular and Metabolic Medicine and Sciences, Faculty of Life Sciences and Medicine, King’s College London London United Kingdom; 4 Division of Endocrinology and Diabetology, Department of Internal Medicine, Medical University of Graz Graz Austria; 5 Department of Endocrinology and Nephrology, Nordsjællands Hospital Hillerød Hillerød Denmark; 6 Institute of Clinical Medicine, University of Copenhagen Copenhagen Denmark; 7 Systems Medicine, School of Medicine, University of Dundee Dundee United Kingdom; 8 Department of Endocrinology, Diabetes, Nutrition, Montpellier University Hospital Montpellier France; 9 Institute of Functional Genomics, University of Montpellier, Centre National de la Recherche Scientifique, Institut National de la Santé et de la Recherche Médicale Montpellier France; 10 Welcome-MRC Institute of Metabolic Science and Department of Medicine, University of Cambridge Cambridge United Kingdom; 11 Department of Internal Medicine, Radboud University Medical Centre Nijmegen Netherlands; 12 Department of Internal Medicine, Division of Endocrinology and Metabolic Disease, Maastricht University Medical Centre Maastricht Netherlands; 13 CARIM School for Cardiovascular Diseases, Maastricht University Maastricht Netherlands; 14 Department of Oncology and Metabolism, University of Sheffield Sheffield United Kingdom; 15 School of Psychology, Institute for Health Transformation, Deakin University Geelong Australia; 16 The Australian Centre for Behavioural Research in Diabetes, Diabetes Victoria Carlton Australia; 17 Diabetes Research Centre, University of Leicester Leicester United Kingdom; 18 Steno Diabetes Center Odense Odense Denmark; 19 See Acknowledgements

**Keywords:** hypoglycemia, diabetes, ecological momentary assessment, smartphone app, content validity, mobile phone

## Abstract

**Background:**

The Hypoglycaemia – MEasurement, ThResholds and ImpaCtS (Hypo-METRICS) smartphone app was developed to investigate the impact of hypoglycemia on daily functioning in adults with type 1 diabetes mellitus or insulin-treated type 2 diabetes mellitus. The app uses ecological momentary assessments, thereby minimizing recall bias and maximizing ecological validity. It was used in the Hypo-METRICS study, a European multicenter observational study wherein participants wore a blinded continuous glucose monitoring device and completed the app assessments 3 times daily for 70 days.

**Objective:**

The 3 aims of the study were to explore the content validity of the app, the acceptability and feasibility of using the app for the duration of the Hypo-METRICS study, and suggestions for future versions of the app.

**Methods:**

Participants who had completed the 70-day Hypo-METRICS study in the United Kingdom were invited to participate in a brief web-based survey and an interview (approximately 1h) to explore their experiences with the app during the Hypo-METRICS study. Thematic analysis of the qualitative data was conducted using both deductive and inductive methods.

**Results:**

A total of 18 adults with diabetes (type 1 diabetes: n=10, 56%; 5/10, 50% female; mean age 47, SD 16 years; type 2 diabetes: n=8, 44%; 2/8, 25% female; mean age 61, SD 9 years) filled out the survey and were interviewed. In exploring content validity, participants overall described the Hypo-METRICS app as relevant, understandable, and comprehensive. In total, 3 themes were derived: hypoglycemia symptoms and experiences are idiosyncratic; it was easy to select ratings on the app, but day-to-day changes were perceived as minimal; and instructions could be improved. Participants offered suggestions for changes or additional questions and functions that could increase engagement and improve content (such as providing more examples with the questions). In exploring acceptability and feasibility, 5 themes were derived: helping science and people with diabetes; easy to fit in, but more flexibility wanted; hypoglycemia delaying responses and increasing completion time; design, functionality, and customizability of the app; and limited change in awareness of symptoms and impact. Participants described using the app as a positive experience overall and as having a possible, although limited, intervention effect in terms of both hypoglycemia awareness and personal impact.

**Conclusions:**

The Hypo-METRICS app shows promise as a new research tool to assess the impact of hypoglycemia on an individual’s daily functioning. Despite suggested improvements, participants’ responses indicated that the app has satisfactory content validity, overall fits in with everyday life, and is suitable for a 10-week research study. Although developed for research purposes, real-time assessments may have clinical value for monitoring and reviewing hypoglycemia symptom awareness and personal impact.

## Introduction

### Background

Hypoglycemia, or low blood glucose, is a common complication of insulin treatment among people with type 1 diabetes mellitus (T1DM) and insulin-treated type 2 diabetes mellitus (T2DM). Hypoglycemia can result in neurocognitive impairment [1] and can be debilitating, adversely affecting multiple aspects of quality of life (eg, work life and relationships) for people with diabetes and their family members [2-4].

The Hypoglycaemia – MEasurement, ThResholds and ImpaCtS (Hypo-METRICS) app was developed for research purposes and uses ecological momentary assessments (EMAs) to capture the impact of hypoglycemia on daily functioning in adults with T1DM or insulin-treated T2DM [5,6]. EMA refers to the repeated assessment of experiences and behavior in real time and in the usual environment of the participants [7]. The Hypo-METRICS app is currently being used for the first time in the Hypo-METRICS clinical study, a European multicenter observational study conducted under the Hypoglycaemia - REdefining SOLutions for better liVEs (Hypo-RESOLVE) project [8]. One aim of the Hypo-METRICS study was to investigate participants’ experiences and the impact of hypoglycemia via a smartphone device 3 times per day (morning, afternoon, and evening *check-ins*) for 70 consecutive days. In addition to their usual method of glucose monitoring, participants wore a blinded continuous glucose monitor (CGM) to record their glucose levels in real time for research purposes. The Hypo-METRICS app enables researchers to investigate the impact of hypoglycemia temporally closer to the occurrence of the episode or episodes, reducing the risk of recall bias and confounding variables. Furthermore, by using smartphones, responses are captured during the participants’ day-to-day lives, not in a hospital or research laboratory setting, thereby optimizing ecological validity [7].

The app was designed iteratively, informed by an independent group of adults with diabetes and a multidisciplinary team of psychologists, diabetologists, health economists, industry partners, and members of the Hypo-RESOLVE consortium patient advisory committee. All stakeholders provided feedback on the content (questions and response options). Content validity is “the degree to which the content of an instrument is an adequate reflection of the construct to be measured” and can be considered in terms of relevance, comprehensiveness, and comprehensibility [9]. Although the content validity of the Hypo-METRICS app was addressed during its development, the COSMIN (Consensus-based Standards for the Selection of Health Measurement Instruments) guidelines for exploring the quality of person-reported outcome measures [9] recommend that new instruments be subjected to additional content validity studies in independent samples.

### Objectives

The first aim of this study was to explore whether the app is “fit for purpose” by investigating its content validity. The second aim was to explore the use of the app in the Hypo-METRICS clinical study in terms of both acceptability (eg, ease of use) and feasibility (eg, completion multiple times per day over 10 wk). The third aim was to explore participants’ suggestions for future versions of the app.

## Methods

### Design

This study was conducted as part of the European Hypo-METRICS clinical study, initiated in October 2020. The study consisted of a short web-based survey (quantitative data) followed by an interview (qualitative data). This combined-method design was used to address the research question. This manuscript followed the COREQ (Consolidated Criteria for Reporting Qualitative Research) checklist [10] (Multimedia Appendix 1). Details of the overall 10-week Hypo-METRICS study have been published previously [5,8].

### The Hypo-METRICS App

The Hypo-METRICS app comprises 29 unique items, forming 7 modules, and uses EMA to examine how hypoglycemia affects daily functioning among adults with T1DM or insulin-treated T2DM [5] (content available in Multimedia Appendix 1). The app was administered via the uMotif Limited platform [11]. Daily functioning includes sleep quality, overall mood, energy levels, negative affect, cognitive functioning, fear of high and low glucose levels, social functioning, and work-related questions. Additional questions capture details of each episode of hypoglycemia (including how it was detected and managed; Multimedia Appendix 1). Participants could only complete each of the check-ins within predefined time intervals (morning: 6 AM to noon; afternoon: noon to 6 PM; evening: 6 PM to midnight). Notifications were sent to remind participants to complete each of the 3 daily check-ins (at 7 AM, 3 PM, and 9 PM). The full details of the Hypo-METRICS app development have been described previously [5].

**Figure 1:**
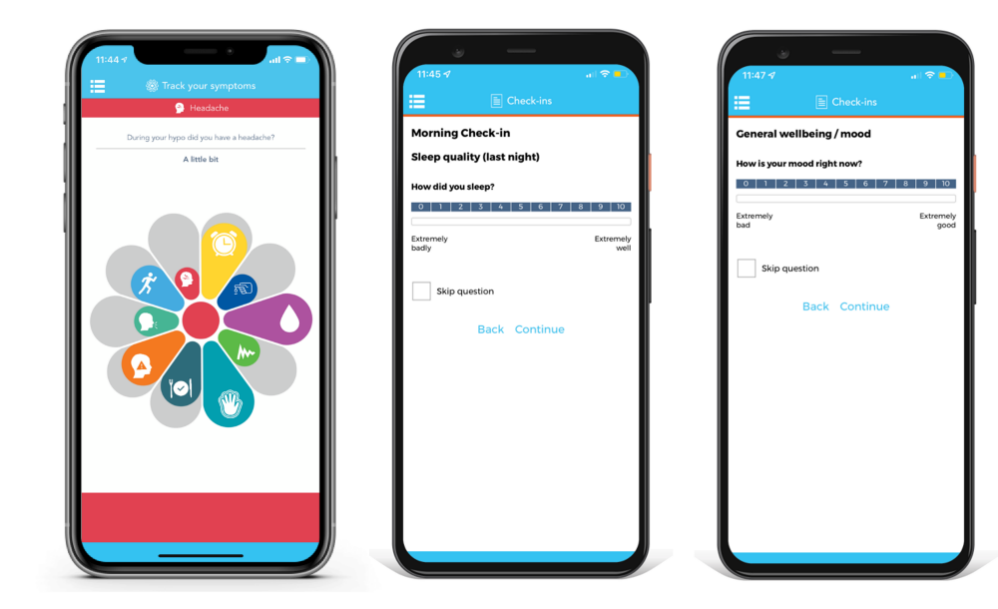
Screenshots of the Hypo-METRICS app on the uMotif Limited platform (left: Motif for recording symptoms; middle and right: check-ins for describing daily functioning and hypoglycemia).

In addition to the check-ins, other features were included in the app for other research purposes, including the *Motif*, which participants were asked to complete after each hypoglycemic episode—and which was used to document hypoglycemic symptoms—by moving the Motif slider to record their response (Figure 1 and Multimedia Appendix 1).

### Study Participants and Recruitment

Participants from clinical sites in the United Kingdom were eligible to participate if they (1) had completed the 70-day Hypo-METRICS clinical study (key inclusion criteria were having T1DM or insulin-treated T2DM and at least one episode of hypoglycemia in the past 3 months [8]), (2) had access to a computer and internet, and (3) were willing to complete a web-based survey and a semistructured interview. Participants were approached (via email or telephone or at the clinic) by the research team and invited to take part. Purposive sampling was used to recruit a heterogeneous subsample of participants (eg, across sexes, types of diabetes, age groups, and check-in completion rates). Participants were not compensated for taking part in the interviews.

### Data Collection

Participants who expressed interest in the interview study received an email including the participant information sheet and provided written consent for participation. Participants’ sociodemographic and clinical characteristics were collected from the Hypo-METRICS REDCap (Research Electronic Data Capture; Vanderbilt University) database. Typically, a few days before their interview, participants completed a brief web-based survey via Qualtrics (Qualtrics International Inc) reflecting on their overall experiences with the Hypo-METRICS app, including (1) motivation to fill in the check-ins, (2) relevance of the check-in questions, (3) understandability of the check-in questions, (4) ease or difficulty of learning how to use the check-ins, (5) design or look of the check-ins, and (6) overall ability to capture the true impact of hypoglycemia (see questions and scales in Multimedia Appendix 1). Each of these questions could be rated on a scale of 0 to 10, with higher ratings indicating a more positive experience. The survey was developed by a core group of the investigators (US, NZ, MB, CH, JS, PC, and FP) and assessed by the remaining coauthors to confirm its face validity.

A semistructured interview guide was developed to explore (1) the content validity of the app and (2) acceptability and feasibility (Multimedia Appendix 1). The interview guide was developed based on similar studies [12], the Mobile App Rating Scale [13], and the COSMIN content validity manuals [9]. All interviews were conducted in English via Zoom (Zoom Video Communications) and audio recorded with the participants’ permission. During the interviews, the participants viewed the app items via a shared screen to help them remember the content. The researcher (US) who led the interviews was a Doctor of Philosophy student (University of Southern Denmark, Odense) at the time of the interviews and received additional training on conducting qualitative research as part of his Doctor of Philosophy training. He had previously conducted cognitive debriefing interviews as part of the translation of the Hypo-METRICS app [5] and received communication training as part of his medical degree. No participants had any previous interactions with US, only knowing that he was a member of the Hypo-METRICS team. The second researcher (NZ), who was present during the interviews, is an experienced qualitative researcher and has had training in qualitative research throughout her Master of Science in Health Psychology and has received further training in qualitative research through the University of Oxford Medical Sociology and Health Experiences Research Group.

In line with the aims and deductive methods of the proposed analysis, the authors followed the procedure recommended by Halcomb and Davidson [14], which consists of extensive field note taking in lieu of full verbatim transcription [15-17]. Researcher US interviewed the participants while NZ took field notes and assisted with prompt questions. After completing the first 2 interviews, NZ and US amended the interview guide based on the initial field notes. After each interview, NZ reviewed the field notes and added to them. Next, US and JLA listened to the audio-recorded interviews one or more time or times to ensure that the field notes represented the interactions in the interview. They added verbatim quotes that were useful to illustrate participants’ feedback and amended the notes if necessary. Recordings and notes were saved on a code-secured external hard drive. The notes and findings were not returned to the participants for comments. During the data collection period, US and NZ met with the wider research team to discuss preliminary findings and amendments to the interview guide. Most of the conversations during the interviews were centered on the 3 check-ins, but participants also shared their experiences with the Motif. If not specified, the descriptions in this paper refer to the check-ins.

### Data Analysis

Descriptive statistics were used to summarize participants’ demographic and clinical characteristics as well as their responses to the web-based survey. These data were presented as numbers and percentages or means and SDs. The notes from the semistructured interviews were uploaded to NVivo (version 11; QSR International) to perform coding and thematic analysis. A deductive approach (theory driven using the research aims described previously) for the initial analysis was followed. Despite this deductive approach (in particular for aim 1), an inductive process to identify themes was used following general principles of the iterative process outlined by Braun and Clarke [18]. An initial coding framework was agreed upon by US and NZ and followed by independent double coding of 20% of the interview notes. Candidate themes were discussed and revised by all authors, and a consensus was reached on the themes to include. Developers’ perspectives on the suitability of the proposed changes were drafted by US and reviewed and amended by the remaining coauthors.

### Ethical Considerations

Ethics approval was obtained from the South Central–Oxford B Research Ethics Committee (20/SC/0112).

## Results

### Overview

Of the 49 participants from the Hypo-METRICS study who were approached, 18 (37%) adults with diabetes were interviewed (T1DM: n=10, 56%; 5/10, 50% female; mean age 47, SD 16 years; T2DM: n=8, 44%; 2/8, 25% female; mean age 61, SD 9 years; Table 1). The interview duration ranged from 29 to 81 (mean 53, SD 15) minutes. All participants completed the brief web-based survey before the interviews.

A total of 8 themes were derived from the interview data and organized by study aim (Textbox 1).

**Table 1:** Participant characteristics.

Characteristic			Type 1 diabetes (n=10)		Type 2 diabetes (n=8)
Sex (female), n (%)			5 (50)		2 (25)
Age (years), mean (SD)			47 (16)		61 (9)
**Recruitment site, n (%)**					
	Cambridge	1 (10)		0 (0)	
	King’s College London	3 (30)		4 (50)	
	Dundee	0 (0)		1 (12)	
	Sheffield	6 (60)		3 (38)	
**Employment, n (%)**					
	Full time	5 (50)		3 (38)	
	Part time	1 (10)		1 (12)	
	Unemployed (but not actively looking for work)	1 (10)		0 (0)	
	Retired	3 (30)		4 (50)	
Impaired awareness of hypoglycemia (Gold score of >4), n (%)			5 (50)		1 (12)
Diabetes duration (y), mean (SD)			26 (16)		16 (8)
HbA_1c_ (mmol/mol), mean (SD)			58.06 (13.54)		57.19 (20.77)
HbA_1c_ (%), mean (SD)			7.46 (1.24)		7.38 (1.90)
**Glucose monitoring modality, n (%)**					
	Flash	5 (50)		2 (25)	
	Finger prick	5 (50)		6 (75)	
**App check-in completion rates (%), mean (SD)**					
	Morning check-ins	85.14 (9.48)		86.43 (10.66)	
	Afternoon check-ins	75.29 (11.35)		86.79 (11.16)	
	Evening check-ins	85.00 (7.66)		94.64 (5.70)	
	Total completion rate	81.81 (8.37)		89.29 (8.76)	
**Web-based survey ratings^a^, mean (SD)**					
	Motivation to use the check-ins	7.80 (1.62)		8.88 (1.13)	
	Relevance of the check-in questions	7.20 (1.93)		8.50 (1.41)	
	Understandability of the check-in questions	7.80 (2.15)		8.75 (1.28)	
	Ease or difficulty of learning how to use the check-ins	7.40 (2.22)		9.38 (0.74)	
	Design or look of the check-ins	7.50 (1.43)		8.75 (1.16)	
	Overall ability to capture the true impact of hypoglycemia	6.60 (1.65)		7.38 (1.60)	

^a^Possible ratings: 0 to 10. Higher ratings indicate more positive experiences. The full questions and response options are available in Multimedia Appendix 1.

**Themes by study aim.**

**Aim 1: exploring content validity**

- Theme 1.1: hypoglycemia symptoms and experiences are idiosyncratic
- Theme 1.2: easy to select ratings on the app, but day-to-day variation was perceived as minimal
- Theme 1.3: instructions could be improved

**Aim 2: exploring the feasibility and acceptability of using the Hypo-METRICS app**

- Theme 2.1: helping science and people with diabetes
- Theme 2.2: easy to fit in, but more flexibility wanted
- Theme 2.3: hypoglycemia delaying responses and increasing completion time
- Theme 2.4: design, functionality, and customizability of the app
- Theme 2.5: limited change in awareness of symptoms and impact

**Aim 3: suggestions for future versions of the app**

### Exploring Content Validity

#### Overview

The mean responses to the web-based survey questions were all >6.50 on the scale from 0 to 10 (Table 1), with individual scores ranging from 3 to 10. Multimedia Appendix 2 provides an overview of the web-based survey topics, together with suggested changes by participants (mentioned during the interviews), followed by the Hypo-METRICS app developers’ comments on the suitability of implementing the suggested changes.

#### Hypoglycemia Symptoms and Experiences Are Idiosyncratic

The quantitative data from the cognitive debriefing survey showed that participants with T1DM and T2DM generally found the check-in questions relevant (mean rating 7.20, SD 1.93 and 8.50, SD 1.41, respectively) and easy to understand (mean rating 7.80, SD 2.15 and 8.75, SD 1.28, respectively).

In terms of daily functioning, participants’ views regarding the relevance of the questions varied. Although some participants could pinpoint certain areas that were not relevant to them (eg, “I certainly have no fear of having a hypo” or “I rarely feel anxious”), generally, they “could see why the questions were being asked” and appreciated their potential relevance to others. Some expressed that relevance was context specific:

[The questions] *weren’t necessarily relevant to me at that particular moment, but they could be if I were* [having]*, or had just had a hypo.*

Participants expressed that it was “important” that the app “covered both mental well-being and physical well-being” and generally that the app “acknowledged” how hypoglycemia can “cause other symptoms like grumpiness or forgetfulness” instead of focusing only on whether it happened. Although most participants mentioned that some of the listed symptoms in the Motif were not personally relevant to them, generally, they considered its content relevant. Most participants could mention at least some symptoms listed on the Motif that they had when experiencing hypoglycemia, but others explained that “it just didn’t capture very many” of their symptoms. One participant explained that some of the symptoms on the Motif seemed quite “severe” and less common.

Overall, when asked about the understandability of the questions, participants found that the questions were “clear,” “easy,” “straightforward,” “self-explanatory,” and “made sense.” A few questions were considered more difficult to interpret or difficult to differentiate, and some participants expressed how they had to use their “gut feeling” or “guess what we mean” and how they felt “worried” that their interpretation was giving the wrong data (see examples in Multimedia Appendix 2). Regarding the Motif, participants generally understood what the symptoms were, but some expressed difficulties in judging whether they experienced a certain symptom—“heart palpitations...I didn’t know if I had them”—and also whether an experienced symptom was due to hypoglycemia or another underlying condition.

When asked whether there were other areas of daily life that were affected by hypoglycemia according to the participants’ own experiences, several participants indicated that the app covered all the relevant areas. For example, a participant stated the following:

I could not think of anything [else], I thought it pretty well covered it. Just saying “how bothersome was it today”...that’s a really good question that sort of covers everything.

Examples of changes suggested by participants to improve content validity are presented in Multimedia Appendix 2.

#### Easy to Select Ratings on the App, but Day-to-Day Variation Perceived as Minimal

For most participants, selecting a score (to represent the impact of hypoglycemia on their daily functioning) was perceived as a “pretty easy” and “quick” task, and they felt “fairly certain” in selecting their responses:

I don’t think it was difficult to choose the answer...there is no right or wrong answer, there is only a personal answer.

The scale from 0 to 10 was described as appropriate. One participant explained their approach to scoring as follows:

I think there was [a] slight tendency to just record how I felt, rather than trying to analyze why I felt like that. But that is probably okay, because it’s for the medical professionals to analyze.

Others had reservations about whether they had understood the meaning of the question sufficiently when providing their scores:

...it was easy enough to put a number in, it was more about what the questions was getting at.

Regarding the following question—“How anxious do you feel right now?”—1 participant said the following:

I’m just not very good at monitoring that myself.

In terms of how the hypoglycemic episode was detected, several participants mentioned how the “I just knew” option was really useful:

That is exactly how you feel, even if you don’t have symptoms.

Participants described difficulties with questions requiring the exact time of day or length of time to be reported.

Participants described that their responses “were fairly similar” and “consistent” and would not necessarily “change from one time to the other” and they just “put the same numbers in.” However, despite responses generally being perceived as “all quite the same,” participants also explained that they could experience small changes if they had hypoglycemia and how episodes could make them “feel more tired” and “irritable” and make it “harder to concentrate,” indicating that they experienced changes in relation to hypoglycemia. A participant explained how they thought they “came to understand the questions better” with time:

...what do you mean by anxiety becomes clearer in your head after doing it for 3 or 4 weeks than it is at day one.

When asked about what would generally reflect a meaningful change on the response scales from 0 to 10, participants expressed that a 1-point change was probably not a substantial change, but it was described that “it might have subconsciously reflected that I was more worried one day or the other.” Although participants generally attempted to answer all questions, the “skip question” option was described as “important” for questions that were not applicable, such as questions related to work for those who were retired.

#### Instructions Could Be Improved

Participants expressed mixed views on the instructions (both verbal and written) given at the study sites. Some found them to be “beneficial” and good “to make sure you knew what was what” and that the instructions had just the right amount of detail without “leading the witness;” others found that they were “left with it” or the instructions were given “quite quickly” and they had to rely on a “trial and error approach.” Some explained that they probably could have “figured out the app eventually” and it was quite “self-explanatory” but that, if no introduction was given, it would have taken longer to become familiar with the app. Several participants explained that more detailed instructions or examples, either given at the study sites or directly provided in the app, would be beneficial to understand the purpose behind the questions more clearly:

In some ways I wasn’t too sure what you would actually get from the information I was giving you—it didn’t seem very sort of precise to me.

I think sometimes I was second guessing what you were getting at.

A participant explained how they would have liked some instructions on when to fill out the Motif:

...should I do it as I’m coming out of the hypo...or when I’m fully recovered?

In addition, several participants did not seem aware that a zoom function was available to ease the use of the slider in the Motif flower.

An overarching point was that, despite most of the questions being generic, that is, asked without a specific reference to hypoglycemia (eg, “How anxious do you feel right now?”), several participants described that they thought they were supposed to answer with hypoglycemia in mind:

...you don’t necessarily know what is due to a hypo and what is due to something else.

...“what is your mood now?” well, it’s so difficult...there are so many external factors...how do I think about that in relation to hypos...and it also depends on where you are in your life.

### Exploring the Feasibility and Acceptability of Using the Hypo-METRICS App

#### Helping Science and People With Diabetes

Participants expressed that their motivation to take part in the study and use the app was driven by their desire to help science, researchers, or other people with diabetes:

I am always interested in taking part in research, because I think it is what you should do.

Some found that their motivation to report details was higher after experiencing hypoglycemia. Using the blinded CGM was mentioned as a motivating factor as it offered participants who did not use it as their usual method of glucose monitoring the opportunity to obtain a more detailed overview of their glucose variations after completing the study. The voucher and Fitbit (given to study participants for taking part in the 10-week Hypo-METRICS study) were not key motivators but considered a nice gesture.

#### Easy to Fit in, but More Flexibility Wanted

Although motivation to use the app over the course of the study generally seemed high, variations occurred. Work or other commitments could make it challenging to find time for every check-in. The afternoon check-in was more frequently mentioned as challenging to fit in when “being busy with the day.” Some found it useful to structure a “routine” around completing the app assessments (eg, “before getting out of bed” or “with meals”). Generally, people who worked from home or who were retired found it easier to find the time to complete the assessments compared with those who worked outside the home or with more variable schedules. This was also supported by the fact that scores on the brief web-based survey were, on average, higher among adults with T2DM (a higher proportion of whom were retired).

Some participants found the check-in timings too “rigid,” and missing a check-in could be associated with “guilt” and “pressure.” A participant expressed that it would be beneficial to make it clearer that it “was not a crime to miss a check-in.” Adding some more flexibility around the timings and a bit of a “buffer beyond the cut-off time” was described as having potential to improve engagement with the app. In contrast, other participants suggested reducing the check-in timings (ie, shortening the time intervals) to increase motivation for timely completion as longer intervals made it “easier to put it off.”

In terms of study duration, some “missed it when it stopped” (ie, when the study ended) and could have continued for longer; particularly, retired participants generally seemed to find it easy to fit in as part of their day. Others reported finding it a bit “monotonous” or becoming “bored” and starting to fill it out in a more “automatic” manner. However, they were still able to “persevere” as they “could understand the sense of why we were doing it.” Most reported that they spent a few minutes completing each check-in (maximum 15 min), which they found acceptable.

#### Hypoglycemia Delaying Responses and Increasing Completion Time

Hypoglycemia could extend the time spent completing check-ins because of the direct effects of the episode (eg, “your brain is working a bit slower”) and because there were more details to report. Many participants expressed that it was necessary to delay the completion of the Motif “maybe 20 minutes” or longer before adding details about symptoms, either because they were “busy trying to treat” their hypoglycemia or because of feeling “disoriented” or “confused” from the hypoglycemic episode itself.

#### Design, Functionality, and Customizability

In terms of functionality and design, the quantitative data (scale from 0-10) showed that participants generally found the check-in functionalities easy to use (T1DM: mean rating 7.40, SD 2.22; T2DM: mean rating 9.38, SD 0.74) and liked the design (T1DM: mean rating 7.50, SD 1.43; T2DM: mean rating 8.75, SD 1.16).

Participants described the design of the app and its functions as “positive,” “straightforward,” “easy,” “fine,” “pretty simple,” “readable,” and “well designed.” Participants who did not consider themselves “particularly tech savvy” said the following:

...even I could manage the app and the check-ins...Assuming someone is able to read and write, I think they are actually very easy to use.

However, some expressed that they did not like the “touch technology” and that a computer keyboard would be easier for them. When asked if they had any suggestions for changes to the design of the check-ins, one participant said the following:

No, I would keep it exactly as it is—it is very simple to use. It’s literally one question on each page, so you don’t get confused by anything and you’re not distracted by anything else...I really liked the design of the app.

Some technical issues were raised, although these were considered minor and did not seem to cause major disruptions to check-in completion, for example, experiences of being asked to complete check-ins that the participants had already submitted and a longer loading time (ie, time to load each page) as the study progressed. Some participants explained how important it was to have study site contacts:

Initially I didn’t know how to do the settings...I’m not a tech man. So, I had to call them and get this thing sorted out. Once I was on the move, I was fine.

Several participants liked the design of the Motif flower, that it “wasn’t just tick boxes,” describing it as “engaging,” “very eye catching,” and “clever.” Some preferred the Motif to the check-ins, particularly when they had multiple hypoglycemic episodes to record. However, several participants described the slider on the Motif as “fiddly” and “chunkier.” One participant commented that they tried to avoid hypoglycemia so they would not need to complete the Motif. Those finding the Motif slider problematic did not seem to know that a zoom option (that made the slider larger) was available. Several participants explained how the Motif setup was very “innovative” and, as the sliders were easy to use, it allowed them to fill out details in the Motif at the time of the episode despite still feeling affected by it.

Participants generally expressed how customizability would be beneficial in terms of both functions (eg, customizing notifications and time intervals) and content (eg, option to remove content that would never be applicable to the individual, such as work productivity for retired participants).

#### Limited Change in Awareness of Symptoms and Impact

Most participants did not report any change in either their perception or experiences of symptoms of hypoglycemia (physical and emotional) or routine treatment of hypoglycemia from using the app. However, some participants experienced changes, both positive and negative. Some of these changes were related specifically to physical symptoms:

...it made me reflect on if I was experiencing symptoms.

...it did make you think “oh goodness yes, actually I did not realise I also had that symptom.”

For some, using the app led to increased awareness of how hypoglycemia affected their emotional well-being:

I thought it was quite nice it was talking about mood and how well you got on with other people and things like that...I know I do get really annoyed and really grumpy, and I would think “oh it’s just me, I’m just a really grumpy person,” but actually...a few times, it is because I’m a bit low or a bit high, so it was really nice “oh, I’m not such a horrible person.”

For others, the change in awareness also extended into how hypoglycemia could affect significant others:

It did make me think about the impact of hypos, not just on me, but on my friends and family as well.

One participant explained a negative emotional impact of using the app and highlighted the importance of informing participants of opportunities for support services if needed:

...doing constant questionnaires about it [diabetes] and thinking about it regularly, I think I got really upset about the long-term health complications. It did really start to get to me over the 10 weeks, and I had to remind myself that was because I was constantly thinking about it due to the app.

### Suggestions for Future Versions of the App

Across each theme, participants made several suggestions for improvements. Multimedia Appendix 2 summarizes them and includes the app developers’ perspectives on the suitability of the proposed changes. The summary follows the structure of the interview guide (ie, within the topics in which they were discussed).

## Discussion

### Principal Findings

This study investigated users’ experiences with the Hypo-METRICS smartphone app, a tool developed for research purposes to explore the impact of hypoglycemia on daily functioning in real life among people with T1DM and insulin-treated T2DM. The app allows for the collection of data during daily life and temporally closer to the hypoglycemic episodes as they occur. Overall, participants seemed to find the content relevant, understandable, and comprehensive, suggesting satisfactory content validity. Responding to questions was generally considered easy, and the response options were appropriate. Some participants indicated that they experienced only minimal fluctuations in ratings over time, querying how useful the thrice daily check-ins would be to the researchers. Some participants suggested that the instructions could be improved to aid their general understanding of the purpose of the study and the meaning of certain questions. Overall, the interviews suggest that participants had a positive experience using the app as part of a research study, and the interviews support the feasibility of investigating the impact of hypoglycemia on daily functioning with multiple daily assessments and use of smartphone technology. The time investment (a few minutes for most participants) required to complete the check-ins was considered acceptable. A few additional functionalities were suggested to further increase motivation and minimize the potential for automatic responses. The interviews also revealed a potential, although limited, intervention effect, with some participants reporting greater awareness of hypoglycemic symptoms as well as greater awareness of the impact of hypoglycemia on their own emotional well-being and on other people.

The interviews yielded suggestions for how to improve the user instructions given by the research staff at the study sites or provided within the app itself. A total of 5 examples of insufficient instructions are highlighted in this paragraph. First, most of the app questions were designed to be generic (eg, “How is your mood right now?”) as it was expected that people would find it challenging to know whether their mood was attributable to hypoglycemia or to other factors (eg, work or sleep [5,19]). However, some participants indicated that they thought the question asked about the impact of hypoglycemia specifically, likely as they knew they were taking part in a study on hypoglycemia. In-app instructions, for example, a brief video when loading the app for the first time, could be considered to instruct participants to respond without reference to hypoglycemia. Second, participants who expressed difficulties in understanding certain questions also indicated that they had to rely on their own interpretation of the questions. A more thorough briefing of the questions, including a statement that a participant’s own interpretation of their thoughts and emotions when answering the questions was expected (ie, that there is no right or wrong answer), could potentially have increased participant trust in their responses. Third, participants expressed concerns about the value of their data as they perceived minimal changes from one check-in to another. Future iterations of the app could include advice to reassure participants that all data are valid and useful regardless of the between-check-in variations in responses (eg, “Please select the score that best describes your experience in the moment. For some questions, you may have experienced little or no change over time, whereas for others, there may be larger changes in experiences. All experiences are valuable, and there are no right or wrong answers”). Fourth, informing participants that the check-in intervals were selected to minimize recall bias and missing occasional responses was okay could potentially have reduced the frustration that participants experienced from the “rigid” time intervals. Previous studies using eye-tracking technology have shown that some participants do not read instructions in detail [20]; thus, future versions of the app could explore the use of in-app videos or more examples in relation to the questions or embedding instructions into the questions or response options as alternative ways of including more guidance to support participants and minimize participant frustration instead of only providing lengthy instructions at study start. Fifth, comments from participants also revealed that, if they understand why the data are being collected and how the data can help them or others, participants will be motivated to put in the effort even for a longer time. As participants described higher motivation to complete the check-ins on days with hypoglycemia, the risk of reporting bias should be explored further (eg, comparing completion rate based on the presence or absence of hypoglycemia).

The interviews also addressed the challenges of relying on self-report when assessing the impact of a condition that acutely affects cognitive functioning. Participants explained difficulties concentrating or focusing on completing the Motif during hypoglycemic episodes. Future iterations of the app could consider exploring cognitive functioning using more objective (ie, not self-report) tasks, as done in other EMA studies [21] (eg, assessing visual-spatial attention and processing speed by selecting matching cards shown on the screen). The cognitive impairment described by participants further suggests that reducing the recall period even further is difficult as the person with diabetes needs to prioritize treatment over answering questions in the Motif. Future studies could consider using CGM-prompted assessments with varying durations from the onset of the hypoglycemic episode to assess the durational effects of both symptomatic and asymptomatic hypoglycemia.

The design of the Hypo-METRICS study was observational, but interviews with users revealed some potential intervention effects of completing the app assessments multiple times daily for 70 consecutive days. Participants explained how being asked about hypoglycemia symptoms made them reflect more on the symptoms they experienced. This effect will be further assessed using the quantitative Hypo-METRICS data collected. These observations align with those of previous pilot studies that used apps to capture hypoglycemic symptoms [22,23]. Existing hypoglycemia awareness training and educational programs [24,25] could consider implementing app-based assessments, such as the Motif, to explore the Motif’s additive effect on restoring awareness. Participants’ descriptions of more atypical symptoms of hypoglycemia suggest a need for customizability in assessing hypoglycemia symptoms. The importance of customizability for participants’ acceptability in EMA studies, in terms of both functions (eg, timing of assessments) and questions (eg, hypoglycemia symptoms), has been similarly described in other EMA studies [26].

Participants in this study described how using the app gave them more insights into how hypoglycemia affects them (eg, their mood) and how it can affect other people, including family and friends. A recent systematic review described how hypoglycemia can negatively affect family members’ emotional well-being and their relationship with the person with diabetes and how greater involvement of family members in clinical care could prevent or reduce conflicts [3]. Participants using the app not only reflected more on but also had more opportunities to discuss the daily impact of hypoglycemia with significant others. For future studies, researchers could consider developing a “family member” version of the app or ask family members to actively take part in responding to the app to explore the potential value for the person with diabetes or the family member.

A recent qualitative study with people with T1DM explored the areas most important to an individual’s quality of life and described how hypoglycemia affects the following domains: relationships and social life, work and studies, leisure and physical activity, everyday life, sleep, sex life, physical health, and mental health [27]. Although some of these may not be suitable to include in an app focused on daily assessments (eg, the subdomain “employment prospects”), others could be considered as amendments for future versions of the Hypo-METRICS app either as separate questions or as changes to or examples within existing questions, including driving, dietary freedom, spontaneity, and relationships or social life. Interestingly, participants in this study made no mention of their “sex life” in the context of hypoglycemia during their interviews. This is generally a topic that people can find uncomfortable or embarrassing to talk about [28,29] and suggests the importance of also exploring the comprehensiveness of questionnaires in written and anonymous formats.

Some interview participants described the importance of studying the impact of hypoglycemia on both physical and psychological aspects, whereas others found the impact of hypoglycemia on aspects of their daily functioning to be limited (eg, “I certainly have no fear of having a hypo”). Past research has consistently shown that severe episodes (requiring assistance from others for recovery) have negative physical and psychological impacts, whereas the findings for self-treated episodes are mixed [2]. Mixed evidence for self-treated episodes might be due to between-person variation, as was also expressed by interview participants in this study. This aligns with recent discussions regarding how some people with diabetes can experience that their mental health is closely related to their glucose fluctuations, whereas for others, they seem unrelated, and therapeutic approaches need to be considered based on individuals’ unmet needs [30]. Longitudinal monitoring of behavior, glycemic values, and mental health could be used to identify individuals’ needs. Although implementation of eHealth in clinical practice has previously proved challenging, app-based tools have the potential to improve self-management, guide scheduling of in-person visits (ie, triage and prioritizing those who need more frequent visits or a referral to psychologists), and reduce costs [31-33]. Furthermore, some people can be hesitant to address mental health issues directly with their health care professionals, and apps might provide a safe opportunity for people to describe their experiences [34].

Recent systematic reviews have concluded that more work is needed to better understand how self-treated hypoglycemia affects the person with diabetes, including domains such as anxiety, mood, and sleep quality [2,35]. Furthermore, the accuracy of effect sizes has been questioned because of recall bias and lack of ecological validity [2,35]. Other app-based studies have focused on the impact of hypoglycemia on domains such as cognitive functioning, physical activity, mood, and diabetes distress [21,36-38]. However, to our knowledge, the Hypo-METRICS app is a unique tool for enabling (1) multiple daily assessments of the impact of hypoglycemia on a broad range of daily functioning domains; (2) detailed daily assessments of person-reported hypoglycemia (including how episodes were detected and managed); and (3) an intensive multiweek (eg, 10-wk) investigation of hypoglycemia awareness and impact, which can be matched to CGM traces. Earlier findings have shown high completion rates and satisfactory psychometric properties of the Hypo-METRICS app [39], and this combined-method approach further supports the content validity, acceptability, and feasibility of the app, enabling innovative future research studies of personalized, real-world, detailed assessments of awareness, management, and impact of hypoglycemia among adults with T1DM and T2DM.

A key strength of this study is that experiences were assessed among participants who had used the Hypo-METRICS app for an extensive period and in the context of their day-to-day lives rather than only in short-duration piloting. The detailed interview guide developed before the interviews ensured overall uniformity in the questions and topics addressed across all the interviews. The purposive sampling strategy enabled balanced representation across sex, age, and type of diabetes; however, as expected, fewer participants with lower app completion (<168/210, <80% check-ins; ie, the participants could complete 3 daily check-ins for 70 days) were recruited. Only participants from the UK sites of the Hypo-METRICS study were included in the interviews, and the transferability of the findings to other non–English-language sites included in the Hypo-METRICS study is limited. Although verbatim transcripts, compared with the notes used in this study, can help other researchers assess the data analysis, others argue that the cross-checking should be from the audio recordings and not from transcripts that may include errors [14]. As participants were answering other questionnaires during the study period in addition to the Hypo-METRICS check-ins and Motif, this may have biased participants’ overall experience. Despite participants perceiving limited variation in their ratings over time, this needs to be examined empirically, and work is required to determine the minimal important differences in app ratings [40].

### Conclusions

This study explored users’ experiences with the Hypo-METRICS app. Overall, the findings suggest that participants had positive experiences using the app; that the content was relevant, understandable, and comprehensive; and that the app is a feasible and acceptable tool to assess the impact of hypoglycemia on daily functioning. For future versions, some modifications and customizability of functions and content could be implemented to increase engagement and content validity. The interviews further revealed a potential intervention effect, suggesting some improved awareness of hypoglycemia symptoms and impact, which warrants further investigation for future research and clinical care. Meanwhile, these data suggest that the Hypo-METRICS app in its present form provides a relevant, acceptable, and feasible new tool for assessing the impact of hypoglycemia on people’s daily lives.

## Appendix

Multimedia Appendix 1 Hypoglycaemia – MEasurement, ThResholds and ImpaCtS app content, web-based survey, interview guide, and COREQ (Consolidated Criteria for Reporting Qualitative Research) checklist.

Multimedia Appendix 2 Participants’ suggestions for future versions of the app by topic and developers’ perspectives on their suitability.

## Abbreviations

CGM: continuous glucose monitor
COREQ: Consolidated Criteria for Reporting Qualitative Research
COSMIN: Consensus-based Standards for the Selection of Health Measurement Instruments
EMA: ecological momentary assessment
Hypo-METRICS: Hypoglycaemia – MEasurement, ThResholds and ImpaCtS
Hypo-RESOLVE: Hypoglycaemia - REdefining SOLutions for better liVEs
REDCap: Research Electronic Data Capture
T1DM: type 1 diabetes mellitus
T2DM: type 2 diabetes mellitus
