# Supplementary material for: Assessing the Content Validity, Acceptability, and Feasibility of the Hypo-METRICS App: Survey and Interview Study
Source: JMIR Diabetes. 2023 Sep 29;8:e42100. doi: 10.2196/42100 (PMC10576226; doi:10.2196/42100)
Supplement: Multimedia Appendix 1 [file diabetes_v8i1e42100_app1.docx]

**Multimedia Appendix 1: Hypo-METRICS app content, web-based survey, interview guide and COREQ checklist”**

**Table S1: Items per module, response options, daily functioning domains, and completion timepoints (for ‘check-ins’) [5]**

| **The three daily check-ins** | | | | | |
| --- | --- | --- | --- | --- | --- |
| **Module names^1^ and items** | **Response option** | **Domains of daily functioning^2^** | **Completion timepoints**  **(‘Check-ins’)** | | |
|  |  |  | **Morning** | **Afternoon** | **Evening** |
| ***Sleep quality module (2 items)*** | | | | | |
| 1. *How well did you sleep?* | *Extremely badly (0) – Extremely well (10)* | *Sleep quality (1/2)* | *x* |  |  |
| 2. *When you woke up how did you feel?* | *Extremely tired (0) – Extremely rested (10)* | *Sleep quality (2/2)* | *x* |  |  |
| ***General well-being module (7 items)*** | | | | | |
| 3. *How is your mood right now?* | *Extremely bad (0) – Extremely good (10)* | *Overall mood (1/1)* | *x* | *x* | *x* |
| 4. *How anxious do you feel right now?^3^* | *Not at all (0) - Extremely (10)* | *Negative affect (1/2)* | *x* | *x* | *x* |
| 5. *How irritable do you feel right now?^3^* | *Not at all (0) - Extremely (10)* | *Negative affect (2/2)* | *x* | *x* | *x* |
| 6. *How is your energy level right now?* | *Extremely low (0) – Extremely high (10)* | *Energy levels (1/1)* | *x* | *x* | *x* |
| 7. *How alert do you feel right now?* | *Not at all (0) - Extremely (10)* | *Cognitive functioning (1/2)* | *x* | *x* | *x* |
| 8. *How well are you able to concentrate right now?* | *Not at all (0) - Extremely (10)* | *Cognitive functioning (2/2)* | *x* | *x* | *x* |
| 8. *How easy was it for you to remember things today?* | *Not at all (0) - Extremely (10)* | *Memory (today) (1/1)* |  |  | *x* |
| ***Fear of hypo-/hyperglycaemia module (4 items)*** | | | | | |
| *10. How worried are you about having a hypo later today?^3^* | *Not at all (0) - Extremely (10)* | *Fear of hypoglycaemia later today (1/1)* | *x* | *x* |  |
| *11. How worried are you about having high blood glucose later today?^3^* | *Not at all (0) - Extremely (10)* | *Fear of hyperglycaemia later today (1/1)* | *x* | *x* |  |
| *12. How worried are you about having a hypo while asleep?^3^* | *Not at all (0) - Extremely (10)* | *Fear of hypoglycaemia while asleep (1/1)* |  |  | *x* |
| *13. How worried are you about having high blood glucose while asleep?^3^* | *Not at all (0) – Extremely (10)* | *Fear of hyperglycaemia while asleep (1/1)* |  |  | *x* |
| ***Social interactions module (1 item)*** | | | | | |
| *14. How well did you get along with other people today?* | *Extremely badly (0) – Extremely well (10)* | *Social functioning (1/1)* |  |  | *x* |
| ***Work and productivity module (4 items)*** | | | | | |
| *15. How many hours did you work today?* | *(Select hours / minute)* | *Hours worked (1/1)* |  |  | *x* |
| *16. How many hours did you miss from work for ANY reason today? [this includes health issues, vacation, holiday, etc.]* | *(Select hours / minute)* | *Hours missed from work (1/1)* |  |  | *x* |
| *17. How many hours did you miss from activities other than work today for ANY reason (e.g. study, housework, shopping, family or leisure activities)?* | *(Select hours / minute)* | *Hours missed from other activities than work (1/1)* |  |  | *x* |
| *18. How productive were you while working today?* | *Extremely unproductive (0) – Extremely productive (10)* | *Productivity (1/1)* |  |  | *x* |
| ***Self-report of hypos while asleep module (8 items)*** | | | | | |
| *19. During the night, did you have a hypo OR take action to prevent a hypo that was about to happen?* | *Yes, No, Not sure* | *NA* | *x* |  |  |
| *20. How many hypos did you have?* | *(Select number)* | *NA* | *x* |  |  |
| *21. At what time did this happen?* | *(Select hour / minute)* | *NA* | *x* |  |  |
| *22. How did you detect your hypo or a hypo that was about to happen? (Select all that apply)* | *- I had symptoms*  *- Someone told me they thought I might be having a hypo*  *- I did a finger prick check (SMBG)*  *- I checked my sensor*  *- My sensor alarmed*  *- I “just knew”*  *- I slept through it – and realised when I woke up*  *- Other:* | *NA* | *x* |  |  |
| *23. What happened? (Select all that apply)* | *- I ate / drank to prevent a hypo*  *- I ate / drank to treat a hypo*  *- I reduced insulin*  *-Someone else gave me something to eat/drink*  *-Someone else gave me glucagon*  *-An ambulance was called*  *-I was admitted to hospital*  *-I took no action*  *-None of the above*  *-Skip questions* | *NA* | *x* |  |  |
| *24. Overall: How bothersome was hypoglycaemia for you last night?* | *Not at all (0) – Extremely (10)* | *NA* | *x* |  |  |
| *25. Overall: How much sleep did you lose due to hypoglycaemia?* | *(Select hour / minute)* | *NA* | *x* |  |  |
| *26. Overall: How worried were you about going back to sleep?* | *Not at all (0) – Extremely (10)* | *NA* | *x* |  |  |
| ***Self-report of daytime hypos module (7 items)*** | | | | | |
| *27. Today, did you have a hypo OR take action to prevent a hypo that was about to happen?* | *Yes, No, Not sure* | *NA* |  |  | *x* |
| *20.1 How many hypos did you have?* | *(Select number)* | *NA* |  |  | *x* |
| *21.1 At what time did this happen?* | *(Select hour / minute)* | *NA* |  |  | *x* |
| *22.1 How did you detect your hypo or a hypo that was about to happen?* | *- I had symptoms*  *- Someone told me they thought I might be having a hypo*  *- I did a finger prick check (SMBG)*  *- I checked my sensor*  *- My sensor alarmed*  *- I “just knew”*  *- Other:* | *NA* |  |  | *x* |
| *23.1 What happened?* | *- I ate / drank to prevent a hypo*  *- I ate / drank to treat a hypo*  *- I reduced insulin*  *-Someone else gave me something to eat/drink*  *-Someone else gave me glucagon*  *-An ambulance was called*  *-I was admitted to hospital*  *-I took no action*  *-None of the above*  *-Skip questions* | *NA* |  |  | *x* |
| *28. Overall: How bothersome was hypoglycaemia for you today?* | *Not at all (0) – Extremely* *(10)* | *NA* |  |  | *x* |
| *29. Overall: How long was it before you were feeling your “usual self” again?* | *(Select hour / minute)* | *NA* |  |  | *x* |
| ^1^Module names refer to the titles used in the app when presenting the items (i.e., what the study participant would see)  ^2^Domains of daily functioning refer to individual items or subscales, developed based on psychometric analyses [39]; for example, the domain ‘Sleep quality’ is a subscale consisting of two individual items, while the domain ‘Overall mood’ consist of just one item. Subscales were created taking the average of the item scores.  ^3^Scores on certain items (anxiety, irritability, and fear related items) were reversed in analyses, so that a higher score indicate improvement (i.e., less anxiety, irritability, or fear). This was to align that an increase in scores on all items would indicate improvement of daily functioning. | | | | | |

**Table S2: Questions in the ‘Motif flower’ function**

| **The Motif flower questions** | ***Response options*** | | | | |
| --- | --- | --- | --- | --- | --- |
|  | ***0*** | ***1*** | ***2*** | ***3*** | ***4*** |
| *1.* *When was your hypo (approximately)?* | *Now* | *15 mins ago* | *30 mins ago* | *1 hour ago* | *>1 hour ago* |
| *2.* *What was your glucose level during your hypo?* | *Not measured* | *<2mmol/L* | *2 to 2.9mmol/L* | *3 to 3.9 mmol/L* | *≥4.0mmol/L* |
| *3.* *During your hypo did you start sweating?* | *Not at all* | *A little bit* | *Somewhat* | *Quite a bit* | *Very much* |
| *4.* *During your hypo did you have heart palpitations?* | *Not at all* | *A little bit* | *Somewhat* | *Quite a bit* | *Very much* |
| *5.* *During your hypo did you start shaking?* | *Not at all* | *A little bit* | *Somewhat* | *Quite a bit* | *Very much* |
| *6.* *During your hypo did you feel hungry?* | *Not at all* | *A little bit* | *Somewhat* | *Quite a bit* | *Very much* |
| *7. During your hypo did you feel confused?* | *Not at all* | *A little bit* | *Somewhat* | *Quite a bit* | *Very much* |
| *8. During your hypo did you have difficulties speaking?* | *Not at all* | *A little bit* | *Somewhat* | *Quite a bit* | *Very much* |
| *9. During your hypo did you find it difficult to coordinate your movements?* | *Not at all* | *A little bit* | *Somewhat* | *Quite a bit* | *Very much* |
| *10. During your hypo did you have a headache?* | *Not at all* | *A little bit* | *Somewhat* | *Quite a bit* | *Very much* |

**Supplementary material: Web-based survey**

Introduction text in Qualtrics:

“Welcome to this short online survey asking about your experiences using the Hypo-METRICS App. These questions will help you to reflect on your experiences prior to the call we have together later. You will in the call be asked to elaborate on your responses in this survey.

As you know, the Hypo-METRICS App contains several separate questionnaires:

1. The 3 daily check-ins (morning, afternoon and evening)
2. The Motif flower with the Hypo specific symptoms
3. The EQ-5D-5L (every day after the evening check-in)
4. The PROMIS sleep questionnaire (weekly)
5. The WPAI work and productivity questionnaire (weekly)

On the next page you will be asked to rate your responses on a 0-10 scale. Since our team have developed the questions in the 3 daily check-ins, these are the focus in this survey.

Please answer every question. If you are not sure which answer to select, please choose the one answer that comes closest to describing your experiences.”

**Please write your Hypo-METRICS ID to continue:**

**Hypo-METRICS ID: ______________**

**Survey items:**

1. ***Overall, on a scale from 0-10, how motivated were you (e.g. did you have the time, energy and desire) to complete the morning, afternoon and evening check-ins?***

Extremely unmotivated (0) – Extremely motivated (10)

1. ***Overall, on a scale from 0-10, how relevant were the questions in the morning, afternoon and evening check-ins to your experiences of hypoglycaemia?***

Extremely irrelevant (0) – Extremely relevant (10)

1. ***Overall, on a scale from 0-10, how easy or difficult were the questions in the morning, afternoon and evening check-ins for you to understand?***

Extremely difficult (0) – Extremely easy (10)

1. ***Overall, on a scale from 0-10, how easy or difficult was it to learn how to use the morning, afternoon and evening check-ins (e.g. finding and filling out the check-ins, adding extra “hypos”, using “skip question” option, moving the slider on the scale, submitting the check-ins etc.)?***

Extremely difficult (0) – Extremely easy (10)

1. ***Overall, on a scale from 0-10, how would you rate the design/look of the morning, afternoon and evening check-ins?***

Extremely bad (0) – Extremely good (10)

1. ***Overall, on a scale from 0-10, how well do you think the morning, afternoon and evening check-ins capture the true impact of hypoglycaemia on your day-to-day life?***

Extremely badly (0) – Extremely well (10)

**Supplementary material: Interview guide**

As seen below the questions from the survey (prior to the interview) have been inserted as part of the interview here. The participants will be asked to elaborate on their responses from the survey.

The interview will start with a general introduction to the purpose of the interview:

“I would like to know more about your experiences with the Hypo-METRICS App, in particular the use of the 3 daily check-ins, and how we can improve future versions of the App. In the interview I will ask about your experiences with the App. As you know the App contains several separate questionnaires:

1. The 3 daily check-ins (morning, afternoon and evening)
2. The Motif flower with the Hypo specific symptoms
3. The EQ-5D-5L (every day after the evening check-in)
4. The PROMIS sleep questionnaire (weekly)
5. The WPAI work and productivity questionnaire (weekly)

Since we have developed the questions in the 3 daily check-ins, this part of the App will be the main focus in this interview. I will make sure to emphasize if my questions are regarding the check-ins or the other questionnaires during the interview. You are welcome to have the PDF with the screenshots of the App open during the interview.

The interview will be audio recorded and used to write a paper that includes your and other participants’ feedback. In this paper, quotes from the interview will be used, but your name will not be linked to these.”

Can you please start out by confirming your Hypo-METRICS ID:”

**Confirm Hypo-METRICS ID: ______________**

**Interview guide – Hypo-METRICS App**

| **Main Questions** | Notes |
| --- | --- |
| **Starter question** |  |
| *Can you tell me about your overall experience of using the Hypo-METRICS morning, afternoon and evening check-ins during this study?*  *(Prompt morning, afternoon and evening separately).* |  |
| **Engagement** |  |
| **Scale:**   1. ***Overall, on a scale from 0-10, how motivated were you (e.g. did you have the time, energy and desire) to complete the morning, afternoon and evening check-ins?*** 2. Extremely unmotivated (0) – Extremely motivated (10) 3. *Can you please elaborate on why you chose this number? What did (not) motivate you? (Not enough time? Energy? Desire?)*   **Prompt questions for the check-ins:**   1. *Did your motivation change over the course of the study?* 2. *How long did it take, usually, to fill out the 3 daily check-ins? Was this amount of time OK for you?* 3. *Could something in the check-ins be changed to increase your motivation? If so, what?*   **Prompt questions for the other questionnaires (not the check-ins):**   1. *How motivated were you to use the other questionnaires in the App? (e.g. the daily EQ-5D-5L, the Motif, the weekly sleep and work/productivity questionnaire)?* |  |
| **Content** |  |
| **Scale:**   1. ***Overall, on a scale from 0-10, how relevant were the questions in the morning, afternoon and evening check-ins to your experiences of hypoglycaemia?*** 2. Extremely irrelevant (0) – Extremely relevant (10) 3. *Can you please elaborate on why you chose this number? What was (ir)relevant?* 4. ***Overall, on a scale from 0-10, how easy or difficult were the questions in the morning, afternoon and evening check-ins for you to understand?*** 5. Extremely difficult (0) – Extremely easy (10) 6. *Can you please elaborate on why you chose this number? What was difficult?*   **Prompt questions for the check-ins:**   1. *How easy was it for you to choose your scores on the different days? Were some questions harder to score than others?* 2. *Do you think that the response options in the check-ins were suitable?* 3. *Would you change any question in the check-ins? Add? Remove?* 4. *Was the wording of the items appropriate?* 5. *Did the response options match the question?* 6. *Are there areas of your day-to-day life that are impacted by hypoglycaemia that we didn’t ask about in the app? Which?* 7. *Were there questions in the check-ins you skipped / did not respond to? If so, which? Why? And how often?* 8. *Did the way you responded to questions change over the course of the study? If so how?* 9. *Did using the check-ins affect the way you experienced hypos (felt symptoms) over the course of the study? If so, how?* 10. *Did using the check-ins affect how you felt about hypos (emotionally) over the course of the study? If so, how?* 11. *Did using the check-ins affect how you treated any hypos over the course of the study? If so, how?*   *How did you determine if a hypo was prevented or not?*   1. *Were there hypos you didn’t report to avoid responding to extra questions in the check-in? If so, why?*   **Prompt questions for the other questionnaires (not the check-ins):**   1. *How relevant to you was the content in the other questionnaires?* 2. *Were there any of the other questionnaires that you skipped / did not respond to? If, so which? Why?* |  |
| **Functionality** |  |
| **Scale**   1. ***Overall, on a scale from 0-10, how easy or difficult was it to learn how to use the morning, afternoon and evening check-ins (e.g. finding and filling out the check-ins, adding extra “hypos”, using “skip question” option, moving the slider on the scale, submitting the check-ins etc.)?*** 2. Extremely difficult (0) – Extremely easy (10) 3. *Can you please elaborate on why you chose this number? What was difficult? What could we improve?*   **Prompt questions for the check-ins:**   1. *Did you experience any technical problems with the check-ins? If so, could you solve the problem? How did you solve it?* 2. *Was it always clear what to do next in the App?* 3. *How did you experience the:*   *The time for the notification/reminder?*  *The “Skip question” option?*  *Option to “Add more hypos” and describe each hypo?*  *The e-mail reminders?*  **Prompt questions for the other questionnaires (not the check-ins):**   1. *Did you find it difficult to use any of the other questionnaires in the App?* 2. *Did you use the ‘diary function’ and the ‘graph section’? Did you find it useful?* |  |
| **Design** |  |
| **Scale:**   1. ***Overall, on a scale from 0-10, how would you rate the design/look of the morning, afternoon and evening check-ins?*** 2. Extremely bad (0) – Extremely good (10) 3. *Can you please elaborate on why you chose this number?*   **Prompt questions for the check-ins:**   1. *Was the size of text, buttons, icons etc. appropriate?* 2. *Do you think the check-ins is clearly structured? Why (not)?*   **Prompt questions for the other questionnaires (not the check-ins):**   1. *Did you like the design/aesthetics of the other questionnaires in the App? What did you (not) like?* |  |
| **Final questions** |  |
| *Final rating:*   1. ***Overall, on a scale from 0-10, how well do you think the morning, afternoon and evening check-ins capture the true impact of hypoglycaemia on your day-to-day life?*** 2. Extremely badly (0) – Extremely well (10)   *Can you please elaborate why you chose this number?*  *Any final comments?* |  |

| **COVID-19 specific questions** | Notes |
| --- | --- |
| *How did the Covid-19 pandemic impact on you and your ability to be part of the Hypo-METRICS study and to use the App?*  *Did the Covid-19 situation impact on your hypoglycaemia? If so, how?* |  |

**Supplementary material:** **COREQ checklist**

**COREQ (COnsolidated criteria for REporting Qualitative research) Checklist**

A checklist of items that should be included in reports of qualitative research. You must report the page number in your manuscript where you consider each of the items listed in this checklist. If you have not included this information, either revise your manuscript accordingly before submitting or note N/A.

| **Topic** | **Item No.** | **Guide Questions/Description** | **Reported on Page No.** |
| --- | --- | --- | --- |
| **Domain 1: Research team and reflexivity** |  |  |  |
| *Personal characteristics* |  |  |  |
| Interviewer/facilitator | 1 | Which author/s conducted the interview or focus group? | 5 |
| Credentials | 2 | What were the researcher’s credentials? E.g. PhD, MD | 5 |
| Occupation | 3 | What was their occupation at the time of the study? | 5 |
| Gender | 4 | Was the researcher male or female? | 5 |
| Experience and training | 5 | What experience or training did the researcher have? | 5 |
| *Relationship with participants* |  |  |  |
| Relationship established | 6 | Was a relationship established prior to study commencement? | 5 |
| Participant knowledge of the interviewer | 7 | What did the participants know about the researcher? e.g. personal goals, reasons for doing the research | 5 |
| Interviewer characteristics | 8 | What characteristics were reported about the inter viewer/facilitator? e.g. Bias, assumptions, reasons and interests in the research topic | 5 |
| **Domain 2: Study design** |  |  |  |
| *Theoretical framework* |  |  |  |
| Methodological orientation and Theory | 9 | What methodological orientation was stated to underpin the study? e.g.  grounded theory, discourse analysis, ethnography, phenomenology, content analysis | 5 |
| *Participant selection* |  |  |  |
| Sampling | 10 | How were participants selected? e.g. purposive, convenience, consecutive, snowball | 5 |
| Method of approach | 11 | How were participants approached? e.g. face-to-face, telephone, mail, email | 5 |
| Sample size | 12 | How many participants were in the study? | 6 |
| Non-participation | 13 | How many people refused to participate or dropped out? Reasons? | 6 |
| *Setting* |  |  |  |
| Setting of data collection | 14 | Where was the data collected? e.g. home, clinic, workplace | 5 |
| Presence of nonparticipants | 15 | Was anyone else present besides the participants and researchers? | 5 |
| Description of sample | 16 | What are the important characteristics of the sample? e.g. demographic data, date | 6 |
| *Data collection* |  |  |  |
| Interview guide | 17 | Were questions, prompts, guides provided by the authors? Was it pilot tested? | 5 |
| Repeat interviews | 18 | Were repeat inter views carried out? If yes, how many? | N/A |
| Audio/visual recording | 19 | Did the research use audio or visual recording to collect the data? | 5 |
| Field notes | 20 | Were field notes made during and/or after the inter view or focus group? | 5 |
| Duration | 21 | What was the duration of the inter views or focus group? | 6 |
| Data saturation | 22 | Was data saturation discussed? | N/A |
| Transcripts returned | 23 | Were transcripts returned to participants for comment and/or correction? | 5 |
| **Domain 3: analysis and findings** |  |  |  |
| *Data analysis* |  |  |  |
| Number of data coders | 24 | How many data coders coded the data? | 5 |
| Description of the coding tree | 25 | Did authors provide a description of the coding tree? | N/A |
| Derivation of themes | 26 | Were themes identified in advance or derived from the data? | 5 |
| Software | 27 | What software, if applicable, was used to manage the data? | 5 |
| Participant checking | 28 | Did participants provide feedback on the findings? | 5 |
| *Reporting* |  |  |  |
| Quotations presented | 29 | Were participant quotations presented to illustrate the themes/findings?  Was each quotation identified? e.g. participant number | 7-14 |
| Data and findings consistent | 30 | Was there consistency between the data presented and the findings? | 7-14 |
| Clarity of major themes | 31 | Were major themes clearly presented in the findings? | 7 |
| Clarity of minor themes | 32 | Is there a description of diverse cases or discussion of minor themes? | 7-17 |

Developed from: Tong A, Sainsbury P, Craig J. Consolidated criteria for reporting qualitative research (COREQ): a 32-item checklist for interviews and focus groups. *International Journal for Quality in Health Care*. 2007. Volume 19, Number 6: pp. 349 – 357

**Once you have completed this checklist, please save a copy and upload it as part of your submission. DO NOT** **include this checklist as part of the main manuscript document. It must be uploaded as a separate file.**
