# Supplementary material for: Assessing the Content Validity, Acceptability, and Feasibility of the Hypo-METRICS App: Survey and Interview Study
Source: JMIR Diabetes. 2023 Sep 29;8:e42100. doi: 10.2196/42100 (PMC10576226; doi:10.2196/42100)
Supplement: Multimedia Appendix 2 [file diabetes_v8i1e42100_app2.docx]

**Multimedia Appendix 2:** Participants’ suggestions for future versions of the app by topic and developers’ perspectives on their suitability.

| **Question and changes suggested by participants** | **Developers’ perspectives on the suitability of changes^a^** |
| --- | --- |
| **Question 1: motivation to use the check-ins (mean rating 8^b^)** | |
| More customizability (flexible time intervals, content, free-text options to allow for elaboration, and choice of platform) | Generally, developers support customizability (eg, option to exclude irrelevant questions and opportunity to use on laptop). For this study, time intervals were chosen to reduce recall bias, and options of free text would increase data analysis and, potentially, participant burden. However, suggestions could be considered for future (clinical) use of the app. |
| Randomize the order of questions (to “make it more engaging”) | Not possible with the current platform but could be considered for future iterations |
| More in-person contact (eg, to maintain the motivation to continue) | Restricted by the COVID-19 pandemic but should be considered in future studies |
| **Question 2: relevance of the check-in questions (mean rating 8)** | |
| Do not assess alertness in the morning | Timing of questions, including alertness, was chosen to minimize recall bias. Future iterations could consider comparing performance-based assessments of cognitive functioning against the existing subjective assessment to ease scoring. |
| Provide definition of “prevented” and “treated” hypoglycemia and make follow-up questions more relevant to the prevented scenario (eg, not asking about the number of hypoglycemic episodes if it was a prevented episode) | Originally, the intention was not to provide a distinct definition of the 2 but rather explore how participants had defined it. However, it was later decided to define hypoglycemia based on symptoms and glucose level (this could be implemented directly in the app to avoid confusion). |
| Include an option to add context to why a person might be fearful of future episodes (“depends on what kind of activities you were intending to do that day”) | Amending context to the “fear of hypoglycemia” question would be relevant (eg, “What worries you?” and provide examples). |
| Add more questions about daily functioning and hypoglycemia (eg, aspects such as driving, physical activity, sports, spontaneity, embarrassment, impact on significant others, and amount of glucose monitoring) | It is important to consider the inclusion of additional relevant questions. The suggested questions could be added as examples to some of the current questions (such as hours lost to activities other than work and how bothersome episodes were). Further debriefing of questions and assessment of participant burden would be required. |
| Add symptoms to the Motif (eg, spotted vision, slurred speaking, urge to urinate, feeling very tired, feeling cold inside, sensation around the mouth, and option to add other conditions with similar symptoms) | All personal experiences with hypoglycemia symptoms are relevant, but it would be difficult to capture them all. It could be an option to add an “Other symptoms” category within the Motif and allow participants to provide the intensity of these symptoms more generally. Alternatively, technology may make it possible for participants to nominate their own unique range of symptoms (from a long list) at study initiation, which they would then rate at each assessment, thereby increasing the personal relevance of the repeated assessment. |
| **Question 3: understandability of the check-in questions (mean rating 8)** | |
| Improve clarity on certain questions (eg, difference between “energy levels” and “alertness”; what “worries,” “sleeping well,” and “activities other than work” mean; and whether “getting along with others” includes partners). | Generally, examples related to each question could address this comment, for example, by providing a “Click here for examples” option for those who find it useful. It would also be important to emphasize in the instructions that individual perspectives might differ and that that is OK; for example, one might worry about a future hypoglycemic episode because of worries of embarrassing themselves, and another person might worry because of the risk of losing consciousness. These are equally valid but different perspectives of worries. |
| Define “work” in the app (eg, whether it refers to paid employment only) | Future versions of the app should include clear definitions of what is considered “work.” |
| Do not include questions that rely on others to respond (eg, for the irritability and social functioning questions) | For the question regarding social functioning, it could be relevant to ask whether the person had been socializing on that day (yesorno) before asking how well they got along with other people. However, “irritability” is a personal experience and does not necessarily rely on interacting with other people (eg, one could feel irritable without interacting with others). Further debriefing could explore how to further optimize these questions. |
| **Question 4: ease or difficulty of learning how to use the check-ins (mean rating 8)** | |
| “Nice to have” functions (eg, link in notifications leading directly to the check-in, option to change the time of notifications, submission confirmation, and study progress bar) | Several of the functions suggested by participants were considered before the study start but could not be implemented within the current platform. These adjustments could be considered in future versions to improve the ease of use. |
| In-app instructions (eg, adding a video and “information” symbol to each question with clarification) | As some of the key problems with the app could have potentially been avoided with more detailed instructions, the idea of in-app instructions is supported by the developers because this would remove reliance on different study site personnel to give (potentially different) instructions. |
| Improve the ease of use of the Motif slider^c^ | It seemed that participants having difficulties with the Motif slider were unaware that a zoom option was available. This emphasizes the need for improvement in the instructions rather than necessarily making changes to the Motif functionality. |
| **Question 5: design or look of the check-ins (mean rating 8)** | |
| Use the Motif design for the check-ins, including a similar color theme | Some participants liked the simplicity of the check-ins, whereas others highlighted the ease of use and more engaging design of the Motif. Therefore, future versions could test the feasibility and acceptability of administering the “check-in” questions via the Motif flower function. |
| **Question 6: overall ability to capture the true impact of hypoglycemia (mean rating 7)** | |
| —^d^ | — |

^a^This column comprises brief summaries of comments on the suitability of the suggested changes from the authors (who also developed the Hypoglycemia Measurement, Thresholds, and Impacts [Hypo-METRICS] app). More details on the Hypo-METRICS app working group can be found elsewhere [5].

^b^Possible rating range: 0-10; higher ratings indicate a more positive experience. Mean ratings with SDs from people with type 1 and type 2 diabetes separately are presented in Table 1.

^c^Note that the questions given in the web-based survey were only regarding the *check-ins*. The Motif was included in the table for ease of access.

^d^No changes suggested were included for question 6 as these were already mentioned previously.
